# A Novel Architecture for Mitigating Botnet Threats in AI-Powered IoT Environments

**DOI:** 10.3390/s26020572

**Published:** 2026-01-14

**Authors:** Vasileios A. Memos, Christos L. Stergiou, Alexandros I. Bermperis, Andreas P. Plageras, Konstantinos E. Psannis

**Affiliations:** Department of Applied Informatics, University of Macedonia, 54636 Thessaloniki, Greece; vmemos@uom.edu.gr (V.A.M.); c.stergiou@uom.edu.gr (C.L.S.); aberberis@uom.edu.gr (A.I.B.); a.plageras@uom.edu.gr (A.P.P.)

**Keywords:** AIoT, botnet, cloud computing, DDoS, DMZ, honeypots, layered architecture, malware, MitM, threat analysis

## Abstract

**Highlights:**

**What are the main findings?**

- The proposed multi-layered architecture enables early detection of botnet activity in AIoT environments.
- Integration of machine learning, sandboxing, and deception techniques improves threat analysis, reduces false positives, and enhances automated response.
- The system demonstrates scalability and resilience, effectively protecting large, distributed AIoT networks, while maintaining operational efficiency.

**What is the implication of the main finding?**

- AIoT systems can operate securely despite the limited computational resources of individual devices, reducing vulnerability to cyber attacks.
- Organizations deploying AIoT solutions can achieve proactive threat management, minimizing downtime and operational disruption from BoT attacks.
- The framework enables threat hunting, forensic investigation, and compliance with privacy regulations, which enhances trust in AIoT technologies.

**Abstract:**

The rapid growth of Artificial Intelligence of Things (AIoT) environments in various sectors has introduced major security challenges, as these smart devices can be exploited by malicious users to form Botnets of Things (BoT). Limited computational resources and weak encryption mechanisms in such devices make them attractive targets for attacks like Distributed Denial of Service (DDoS), Man-in-the-Middle (MitM), and malware distribution. In this paper, we propose a novel multi-layered architecture to mitigate BoT threats in AIoT environments. The system leverages edge traffic inspection, sandboxing, and machine learning techniques to analyze, detect, and prevent suspicious behavior, while uses centralized monitoring and response automation to ensure rapid mitigation. Experimental results demonstrate the necessity and superiority over or parallel to existing models, providing an early detection of botnet activity, reduced false positives, improved forensic capabilities, and scalable protection for large-scale AIoT areas. Overall, this solution delivers a comprehensive, resilient, and proactive framework to protect AIoT assets from evolving cyber threats.

## 1. Introduction

Today, it is fact that Internet of Things (IoT) environments can be converted to a BoT by malicious users who want to intrude into such networks, e.g., an industrial IoT, an educational IoT, a healthcare IoT, etc. [1]. Especially, although the corresponding IoT-based environments with the integration and use of Artificial Intelligence (AI) can become smarter, this evolution also introduces increasing security risks [2]. After the intrusion into the smart devices of these AIoT environments, malicious users can make all the AI-powered “things” (e.g., sensors, actuators, machines, etc.) of them to operate as dynamic autonomous bots (robots) for malicious purposes [3].

The fundamental problem arises from the fact that strong encryption algorithms demand more resources than IoT devices can typically provide. As a result, IoT devices along with wireless actuators and sensors often rely on weaker encryption methods due to their severe limitations in memory, processing power, and battery life. This lack of robust security makes them attractive targets for black-hat hackers, who can exploit vulnerabilities to launch various attacks including DDoS attacks, MitM attacks, and malware distribution [4].

In this research work, we propose a novel multi-layered architecture for mitigating botnet threats, especially those that applied in smart AIoT environments, such as industry, healthcare, agriculture, transportation, etc. Τhis architecture is designed to create a secure and resilient environment for AI-powered IoT operations. It utilizes multiple layers of protection to separate the external network from internal systems and devices, ensuring that only verified and safe connection attempts and data can pass through. At the edge, the incoming traffic is inspected and validated in order to be permitted into the core AIoT network, while suspicious incoming attempts are redirected to isolated and controlled virtual environments for deeper analysis leveraging machine learning (ML) techniques. Our proposed system architecture includes sophisticated components that monitor, analyze, and learn from potential threats, and thus, enables proactive defense and different scalable security measures. This centralized monitoring and automated response mechanisms ensure the rapid detection and mitigation of various cyber attacks, while deception and sandboxing techniques offer significant knowledge about suspicious and malicious behaviors, protecting the real assets of the AIoT area.

Overall, this approach combines prevention, detection, and response in a coordinated way to maintain strong security and operational integrity. The experimental results validate the functionality and effectiveness of our novel scalable architecture for detecting and mitigating BoT threats in AIoT environments. Specifically, when qualitatively compared with other state-of-the-art models, our proposed architecture presents superiority due to the following advantages:

✓ Stronger early detection of botnet/BoT activity.
✓ Reduced false positives and more actionable alerts.
✓ Faster, safer mitigation and containment.
✓ Enhanced forensic analysis and threat intelligence capability.

The rest of this paper is structured as follows: Section 2 cites the related literature review; Section 3 presents the most crucial challenges and risks of the IoT transition to BoT networks; Section 4 provides a thorough description of our proposed architecture; Section 5 includes the experimental setup of our testbed; Section 6 presents the basic experimental results; Section 7 provides a comparative analysis of the proposed architecture with representative state-of-the-art models; and finally, Section 8 concludes the paper providing some potential future directions.

## 2. Related Work

There are many research papers that address the challenges of mitigating botnet threats in IoT and AIoT environments. Elijah et al. [5] trace the development from conventional machine learning (Decision Tree—DT, Support Vector Machine—SVM, Random Forest—RF) to deep learning (CNN, RNN/LSTM, autoencoders) and hybrid paradigms (ensembles, federated/meta-learning), positioning AI-driven intrusion detection and prevention systems (IDPS) as a response to next-generation network (NGN) security complexity (5G, IoT, edge, SDN). Major intrusion detection system (IDS) datasets (NSL-KDD, CICIDS2017, UNSW-NB15, Bot-IoT, IoT-23, TON_IoT) are critically reviewed, and representativeness gaps and operationalization challenges in NGN contexts are noted. The lack of datasets, poor generalization across heterogeneous domains, computational overhead at the edge, problems in adversarial robustness, limited explainability, and privacy risks are all highlighted in the paper. In order to satisfy NGN needs, the authors argue for lightweight yet accurate models, federated frameworks that protect privacy, and closer integration of automated prevention with detection. The review emphasizes realistic NGN datasets and resilience-focused evaluation (adversarial testing, explainability metrics) as prerequisites for reliable deployment, and it clarifies where graph-based models, continual learning, and federated setups fit by synthesizing approaches across SDN, IoT, edge, cloud/5G, and cloud environments.

In their thorough assessment, Menon et al. [6] map the tri-level AIoT computing stack (edge–fog–cloud) and layered IoT/AIoT architectures (3-, 5-, and 7-layer variations plus security/edge extensions). It describes the responsibilities of ML and deep learning (DL) in IoT security (authentication, access control, anomaly/IDS, DDoS mitigation, malware analysis) and places enabling technologies like blockchain, 6G, federated learning, and hyperdimensional computing in the context of devices with limited resources. In this study, supervised, unsupervised, and semi-supervised reinforcement learning (RL) techniques using DL families (CNN, RNN/LSTM, transformers; autoencoders/DBN/RBM; GANs; DRL) are compared. In addition to highlighting trade-offs between SVM and RF, cluster formation restrictions in K-means, and RL convergence/state-transition challenges in IoT scenarios, the survey emphasizes unsupervised learning for zero-day detection in IoT and deep reinforcement learning (DRL) for dynamic policies. Interoperability, scalability, energy limits, privacy/security, heterogeneous data analytics, and “butterfly effect” problems in ML/DL for IoT are all listed in this study. To balance accuracy with interpretability and efficiency in practical deployments, it requires explainable AI (XAI), self-supervised/transfer learning, lightweight accelerators, and neuromorphic/edge-oriented designs.

IoT security is organized around layered architectures (perception, network, transport, application, and business) by Hassan et al. [7]. They map key security features (identity, confidentiality, integrity, availability, and interoperability) to the limitations of each layer (power, memory, protocol heterogeneity) and highlight the necessity of end-to-end, context-aware controls. Jamming, spoofing, Sybil/MITM, wormhole/sinkhole, replay, DDoS, code injection/malware, and other common threats are surveyed in this work, which highlights how traditional signature-based defenses struggle with zero-day patterns in diverse IoT environments. Supply chain exposure and cross-layer risks make compound detection challenging. The work promotes RL for adaptive mitigation under changing traffic and device conditions, federated learning for privacy-preserving model training, and hybrid ML/DL intrusion detection. It emphasizes data governance and explainability as essential safeguards for reliable adoption in safety-critical IoT areas.

Furthermore, Afrin et al. [8] place AI-driven IoT security into Governance, Risk, and Compliance (GRC) frameworks, emphasizing auditability across distributed IoT pipelines, data provenance, and model accountability. The conflict between quick machine learning iterations and legal requirements (privacy, safety, and dependability) in many jurisdictions is brought to light in this work. Model risk management (validation, drift monitoring, red-teaming), privacy-preserving analytics (FL, differential privacy), and policy-aligned explainability to support automated judgments are among the suggested controls. In AIoT situations, it emphasizes standardized incident response and evidence gathering. In order to avoid misconfiguration and silent failure modes in edge deployments, recommendations focus on cross-functional processes—security engineering, data stewardship, legal/compliance—with a focus on documentation, reproducibility, and lifecycle governance.

In their study of ML/DL IDS for IoT traffic, Wang et al. [9] compare DL architectures (CNN for spatial packet characteristics, LSTM for temporal sequences, hybrid AE-CNN stacks) with traditional classifiers (SVM, RF, NB, KNN). This research work addresses label scarcity and feature engineering costs, while also, highlighting performance improvements on IoT-based datasets. To accommodate microcontroller-class devices, practical deployment options include streaming minibatch updates, distillation, and model pruning/quantization. The study proposes adaptive pipelines under resource constraints and compares cloud-offloading with edge inference. There are restrictions on reported advantages related to adversarial susceptibility, maintenance overhead, and generalization to unseen devices/topologies. The authors support benchmarking procedures and dataset curation that are compatible with diverse IoT contexts.

Moreover, Bibi et al. [10] focus on volumetric and protocol/application-layer DDoS in IoT and suggest edge-centric detection utilizing CNN/LSTM hybrids and feature-engineered flow statistics with the goal of quick triage and local mitigation prior to backbone congestion. This work investigates cooperative edge gateways to share signals without raw data exchange (privacy-preserving coordination) and reinforcement learning (RL) to adjust thresholds/policies under drift. Robustness to traffic mutation and latency/accuracy trade-offs are evaluated. Results indicate that co-designing models using edge telemetry and lightweight inference significantly reduces false positives and improves time-to-mitigation, supporting robust smart-city and industrial IoT applications.

In order to safeguard IoT data both in motion and at rest, Mishra [11] describes an integrated stack that combines cryptographic primitives (lightweight encryption/authentication), anomaly based IDS, and policy enforcement. AI orchestrates detection and adaptive responses throughout the pipeline. It describes how dynamic key rotation and access revocation are triggered by DL models (such as autoencoders and LSTM) monitoring telemetry for deviations. The framework balances overheads by focusing on restricted devices with modular components and edge/offload possibilities. Higher detection rates with controllable latency and energy profiles in testbeds are among the reported benefits. In order to maintain performance during device lifecycles, the author emphasizes interoperability with diverse IoT stacks and promotes explainability and federated retraining.

Finally, in order to identify cyberattacks that target virtual machines in private cloud infrastructures, Latesh Kumar et al. [12] provide an AI-powered threat prediction and protection (TPP) algorithm based on a correlational recurrent neural network (CorrNet). In order to detect suspicious patterns in real time, the method makes use of various data sources (IP addresses, ports, timestamps, and OS logs) and correlates them. As a dual autoencoder, CorrNet learns joint representations from two different perspectives of the data (threats and vulnerabilities). Training maximizes correlation between hidden representations while minimizing reconstruction and cross-reconstruction errors. The system incorporates modules (Action, Expect, Log) that keep an eye on user behavior, apps, ports, and scripts, documenting any irregularities in specific forensic files (like Attackers_Dirty_Play_Ground). A cloud-based payment gateway set up on a university campus served as the method’s validation. Comparative scenarios (firewall + TPP, firewall only, TPP only) showed that the combined strategy outperformed conventional defenses by achieving 92.5% accuracy in predicting and blocking threats. Malicious requests were blocked and forensic evidence was preserved by the system’s successful detection of traffic spikes and OS-level anomalies. The paper emphasizes how correlation-based analysis with CorrNet may improve cybersecurity in financial cloud systems by providing forensic capabilities, real-time detection, and flexibility against sophisticated threats like botnets and ransomware. The framework improves resilience and reliability in key infrastructures by fusing AI with traditional tools (like Nmap and Wireshark).

Our proposed system architecture differs from prior conventional AIoT/IoT IDS frameworks in many ways. First, unlike many existing studies that rely on single-protocol or homogeneous IoT environments, our model incorporates heterogeneous protocol stacks and device profiles, combining MQTT (with topic-level ACLs and mTLS), CoAP over UDP, HTTP, and SSH endpoints across emulated legacy and modern devices. This design reflects the protocol diversity and mixed trust environments commonly observed in real AIoT deployments. Second, the integration of a middleware layer enables bidirectional control logic and orchestration between devices and the Edge Gateway (EGW). Such application-level control workflows, which are centralized to AIoT systems, are rarely modeled in prior related research works. Third, our architecture provides an end-to-end edge-to-cloud security pipeline that combines a multi-stage detection (signature-based, unsupervised, and supervised techniques), feature-level explainability, and automated SOAR-based mitigation. This feature moves beyond the detection-only approaches and enables actionable, low-latency responses and improved forensic visibility. Finally, our proposed model introduces realistic variability in benign traffic through randomized telemetry schedules, firmware checks, and administrative activity, reducing excessively clean separability and better reflecting operational noise often absent from controlled benchmarks. Table 1 provides a comparative overview of the proposed architecture against representative AIoT security and IDS frameworks from the literature.

## 3. Challenges and Risks

There are many types of threats that should be addressed by the scientific community, as they raise multiple security and privacy issues [13]. IoT deployments increase these risks because of their decentralized operation, continuous data exchange and large-scale connectivity. Following are the most common threats and challenges derived by AIoT.

### 3.1. Security Challenges

#### 3.1.1. Expanded Attack Surface

The number of possible access points for cyber attackers has significantly increased due to the growth of billions of IoT devices. Every connected device, be it an autonomous car, wearable health monitor, smart thermostat, or industrial sensor, represents a node that could be compromised if not well secured. Because of this wide and diverse environment, the basic security frameworks are insufficient [14].

This problem becomes more critical due to the use of AI-powered IoT networks. Although these networks increase automation and efficiency, they make the architecture and its operations much more complex. AIoT-based devices collect massive volumes of data, and therefore, any compromise in data integrity can result in poor decision-making, system failures, or even physical damage of infrastructure assets.

Furthermore, endpoints and network behaviors can change rapidly in AIoT environments. As a result, the maintenance and control of all devices becomes more challenging, especially when they are distributed across multiple regions and managed by various stakeholders. Attackers can exploit these blind spots and intrude into undetected networks, leveraging techniques and methods such as adversarial Machine Learning (ML), spoofing, or internal reconnaissance [15].

#### 3.1.2. Weak Device Security

The massive use of mobile devices and gadgets with insufficient security features is a significant security flaw. Attackers can easily target them, since such devices do not use advanced security mechanisms. It is fact that many AIoT devices come with default passwords, outdated apps, and low computing power. Such weaknesses can be exploited by specialized botnets like the Mirai which executed large DDoS attacks. Mirai was able to take control of thousands of devices and transform them into a series of bots by finding on the internet devices with default credentials [16].

The issue is made worse by a number of factors, including a lack of standardization because IoT devices are made by a variety of manufacturers, many of whom do not adhere to consistent security standards; a lack of user awareness because users frequently fail to update firmware or change default settings, leaving devices vulnerable; a lack of resources because many IoT devices have limited computing power, making it challenging to implement strong encryption or real-time threat detection; and a long lifespan and poor maintenance because devices can go years without updates, resulting in long-term vulnerabilities. These flaws can also be used by attackers to steal data, penetrate networks, and generate large-scale botnets [17].

#### 3.1.3. Adversarial Attacks on AI Models

Adversarial attacks have surfaced last years, since AI is being integrated into the IoT. Such attacks take advantage of weaknesses in AI models, through specific inputs, by interfering with their decision making process. This fact is achieved by the use of specific improper inputs that can lead an AI model to make incorrect or even harmful decisions. Thus, autonomous systems that are integrated into smart cars, robotics, and medical equipment can directly affect human safety, privacy, and physical operations [18].

These attacks often involve minor alterations to input data (such as pictures, sensor readings, or signals) that are undetectable to humans but extremely disruptive to AI models are frequently subtle and challenging to identify. Most of such models are not trained to handle adversarial scenarios, making them vulnerable to handle input data and understand how decisions are made [19].

#### 3.1.4. Data Integrity Attacks

Data integrity is crucial for AIoT-based devices. AI models mostly rely on the constant streams of data from IoT sensor, in order to make choices in real time, optimize operations, and maintain security. However, attackers can seriously decision making and operational safety by altering sensor data [20]. Because they take advantage of the confidence that AI systems have in sensor data, these attacks are especially harmful. No matter how sophisticated the AI is, the output is unreliable if the input is compromised [21].

#### 3.1.5. Insider Threats

Insider threats can take advantage of their access to sensitive AIoT data, such as real-time sensor or behavior logs, training datasets and proprietary models and system configurations that can disable controls or introduce vulnerabilities [10,22].

#### 3.1.6. Scalability of Security Solutions

AIoT networks are large, distributed and dynamic, often including thousands or even millions of devices in places like hospitals, homes, smart cities, and factories. Because of this size and complexity, traditional centralized security systems fail to respond to their changing needs. Although centralized systems work well in smaller or static networks, they become less effective in AIoT environments due to limited adaptability, bandwidth issues and hardware resource constraints [23].

### 3.2. Privacy Challenges

#### 3.2.1. Massive Data Collection

IoT devices continuously collect large amounts of personal information, such as location data, health data, voice recordings, sleep patterns, and daily routines. This data becomes even more meaningful when combined with AI, because AI can analyze and reveal sensitive details such as emotions, health problems, or even predict future behavior. This can raise serious privacy concerns, especially when people are not aware of how much information is being collected [6].

#### 3.2.2. Surveillance & Profiling

AI algorithms can collect and analyze IoT data to generate detailed user profiles including habits and choices. This feature can cause a potential of digital surveillance by governments and malicious users to track people. The risk is that these profiles could be misused for unfair, manipulative, or illegal monitoring, turning the everyday IoT devices into spying tools without users’ permission [24].

#### 3.2.3. Lack of User Awareness and Control

Most people are not aware of what data their AIoT devices collect how long it is stored or how AI models process it. This happens because device settings and privacy rules are often not clear, while AI-driven actions from such smart devices often lack justification, which makes it hard for users to understand or challenge them [25].

#### 3.2.4. Data Ownership and Consent

The ownership of the data produced by AIoT devices is often unclear. This uncertainty makes data rights more complicated, especially when AI systems use the data for selling services, personalizing content, or training models. It is becoming less clear who owns the data produced by IoT devices—is it the cloud service provider, the user, or the device maker Consent processes are often weak or missing, leaving users with limited control on how their data is used, shared, or sold, especially in AI-based applications where data is continuously reused [12].

#### 3.2.5. Re-Identification Risks

AI systems can often identify people again by looking at patterns and connections, even when IoT data has been anonymized. For instance, the use of smart home devices can reveal everyday human habits, making it possible to figure out routines, identities, or household composition. This fact poses a serious risk to data privacy and makes anonymization less effective, especially when datasets are shared or sold to third parties [26].

#### 3.2.6. Cross-Border Data Transfers

AIoT devices often send data to cloud servers in different countries, which creates legal and regulatory issues. Privacy laws like the California Consumer Privacy Act (CCPA) in the United States or General Data Protection Regulation (GDPR) in Europe can conflict with these cross-border data flows. When data is processed in multiple countries with different rules for user rights, data protection, and consent, it is hard to ensure compliance. This fact can lead to a broken privacy system that is difficult to manage and maintain [27].

## 4. Proposed Scenario

Our proposed scenario includes the following main components:

- 2 robust firewalls that form a demilitarized zone (DMZ)—both an External Firewall (EFW) and an Internal Firewall (IFW): The first one, EFW, is responsible for serving as the first line of defense at the network perimeter, performing packet filtering, Network Address Translation (NAT) and DDoS mitigation, GeoIP blocking, and access control to limit incoming traffic to authorized protocols and endpoints. The second one, IFW, is responsible to separate the DMZ from the Industrial AIoT network, enforcing strict zone-to-zone policies, protocol whitelisting, micro-segmentation, and deep packet inspection (DPI) for industrial traffic (e.g., Modbus, OPC-UA).
- Edge Gateway (EGW): Positioned within the DMZ and acting as an intelligent inspection and decision layer that performs mTLS/CRL validation, protocol sanity checks, rate limiting, telemetry collection, and dynamic security enforcement (e.g., IP blocking, alert generation).
- Internal Middleware Server (IMWS): Also located in the DMZ, it handles validated and clean traffic from the EGW, performing additional application-level processing, message brokering, and integration with backend industrial or AIoT systems.
- Cloud Proxy Server (CPS): Receives shadow copies of suspicious or unknown traffic flows from the EGW, applies routing rules, and directs them either to the honeypot farm or to the IMWS based on initial heuristics and reputation scores.
- Honeypot Farm (2-tier Deception Layer) within a virtual private cloud (VPC): A set of isolated, containerized environments (low- and high-interaction honeypots) that emulate real IoT devices to capture and study attacker behavior, exploit attempts, and payloads in a controlled environment.
- Sandbox Endpoint (SEP): Located inside the VPC, SEP executes captured binaries or commands from the honeypots in a controlled sandbox to analyze dynamic behaviors, file system changes, and network callbacks, while ensuring isolation and containment.
- Threat Analysis Server (TAS): Hosts the ML-based analysis pipeline that correlates data from honeypots, sandbox, and edge telemetry to produce threat scores and behavioral insights using hybrid detection models (signature-, rule-, and ML-based).
- Security Orchestration, Automation, and Response Engine Server (SOARES): Automates mitigation actions and policy updates (e.g., blocking, quarantining, certificate revocation), orchestrates incident response, and provides feedback to the EGW and TAS for adaptive learning and continuous security improvement.
- Next-Generation Security Information and Event Management System (NGSIEMS): Collects, aggregates, and visualizes alerts, logs, and incidents from all components, supporting centralized monitoring, forensic analysis, and threat intelligence integration.

Table 2 summarizes the main architectural components and their roles within the proposed AIoT security framework.

**Figure 1:**
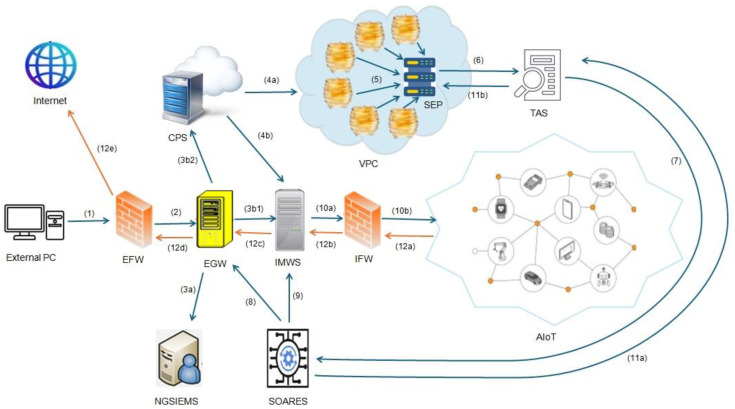
The proposed architecture. The numbered arrows (1–12) and lettered flows (a–d) denote the main communication and data-processing steps between the system components, as described in Section 4.

More specifically, as illustrated in Figure 1, an external PC—which can be either a legitimate user or an attacker—initiates a connection request (case 1) to a device inside the AIoT environment. This request first arrives at the EGW (case 2), which operates as more than a simple router as it performs several preliminary security and validation checks. Such checks include a series of procedures, such as mutual TLS (mTLS) certificate validation, client certificate revocation list (CRL) verification, IP reputation and geolocation, protocol sanity checks for header integrity and length validation, and rate limiting methods.

The EGW maintains, in its database, real-time metrics, events, logs, and traces (MELT) data, such as number of connection attempts, failed authentication requests, and IP reputation score, enabling it to define thresholds and respond quickly. For example, the EGW may classify a sender as suspicious if there are more than 10 connection requests per minute or 5 failed authentication attempts within 60 s. In such cases, the EGW may temporarily block the IP address and/or raise an alert.

For further hardening of this architecture, the EGW is deployed in the middle of two robust firewalls, providing enhanced multi-layered security. The first one is an EFW, which is located at the boundary between the Internet and the edge zone, and is responsible for basic packet filtering, access control, and NAT/DDoS protection. It allows only connections that use specific and authorized communication protocols (e.g., HTTPS/TLS, MQTTs, CoAPs) to approach the EGW. Additional features, such as GeoIP blocking and threat intelligence, can also be applied to reduce the exposure surface. The second one is an IFW, which is located between the EGW and the AIoT network, and is responsible for enforcing zone-to-zone communication control and micro-segmentation. It allows only pre-approved protocols and endpoints (e.g., MQTT over TLS toward authorized brokers, or OPC-UA connections to specific PLCs). Furthermore, it may employ Deep Packet Inspection (DPI) for industrial protocols such as Modbus, PROFINET, or EtherCAT, providing an additional layer of protection for critical industrial assets.

In other words, the EGW is positioned in a DMZ between the two firewalls (EFW and IFW), and acts as an intelligent inspection and decision layer. In addition to performing mTLS and CRL validations, it monitors telemetry, applies behavioral rules (e.g., rate thresholds, repeated failures), and may dynamically update firewall rules or notify the SIEM/IDPS system if suspicious behavior is detected. This layer effectively bridges external connectivity with local control and security intelligence.

Specifically, if the EGW clearly indicate malicious activity due to, for example, a blacklist IP, an invalid cert, or malformed packets, it declines the connection request and logs the event to the NGSIEMS (case 3a) which also acts as an IDPS. On the other hand, if the connection request seems to be normal, it can either be forwarded to the IMWS (case 3b1), or in the case of unknown or suspicious flows, it can be forwarded to the cloud analysis pipeline (case 3b2) while allowing high-confidence clean flows to proceed with a minimal latency. For such suspicious or abnormal flows, the EGW creates a shadow copy of the connection request (mirroring process), which is a sanitized payload, in order to preserve privacy, with additional metadata such as the source IP, the destination device ID, the timestamp, the protocol, the headers, the payload hash, and the reputation score from the EGW’s heuristics. If the payload contains potentially sensitive personal data, the EGW sends only hashed identifiers or sampled bytes.

The shadow copy is sent to a CPS (case 3b2) that acts as a decision-making hub by applying simple routing rules, such as newly seen IPs or raw gateway reputation score, and hence, it determines whether the flow will be routed into the honeypot farm (honeynet) (case 4a) or to the IMWS (case 4b). Therefore, the normal flows are forwarded directly to the IMWS, while the suspicious flows are routed to a 2-tier deception layer, which is a composition of specialized honeypots that have been configured to mirror the real IoT-based devices so as to trap potential attackers.

The 2-tier layer includes both low-interaction honeypots and high-interaction honeypots. The first ones respond to superficial probes and quickly collect brute-force credentials, scanning patterns, and script-kiddie behavior, while the second ones run complete firmware or full service stacks inside well-structured containers or virtual machines (VMs) that mimic the actual device firmware, API endpoints, and timing features, capturing detailed exploitation attempts, persistence mechanisms, file system changes, spawned processes, and abnormal behaviors. All honeypots are deployed in an isolated VPC.

This subnet has a strict policy since it does not allow direct outbound internet access, except through a tightly controlled and monitored SEP that prevents attackers from jumping to the internet via these honeypot machines. Specifically, the captured values, such as packets (PCAPs), commands’ sequence, attempted uploads, dropped binaries, etc., are forwarded to the SEP (case 5) for dynamic analysis. The SEP executes suspicious files in a scalable controlled environment and records any behavioral features like system calls, file writes, registry or config changes, domain name system (DNS) and network callbacks, use of suspicious APIs, and persistent connection attempts. After this process, the SEP returns a behavioral vector and raw logs to the TAS (case 6) which is empowered with ML engine.

The TAS collects and organizes the metadata, honeypot logs, sandbox behaviors, and edge telemetry into a time-series database and object store. By using feature extraction, the TAS converts the raw data into meaningful input vectors: network features (average packet size, inter-packet times, byte entropy, session duration), authentication features (failed login ratio, username variance), sandbox features (syscall counts, external domain callbacks, files dropped, etc.), and device context (model, firmware version, last patch update). The analysis pipeline uses a hybrid ensemble approach: signature detection, rule-based heuristics, unsupervised anomaly detection, and supervised classification. Example unsupervised models include autoencoders for telemetry sequences and long short-term memory (LSTM) for session behavior; supervised models might be RF or eXtreme Gradient Boosting (XGBoost) trained on labeled malicious and benign sessions. The engine outputs threat score in the range 0 to 1 [0, 1].

The decision making follows specific thresholds: scores > 0.9 may be treated as “malicious” and enable immediate mitigation; scores between 0.6 and 0.9 [0.6, 0.9) may be “suspicious” and result in quarantine and human review; scores below 0.6 [0, 0.6) are treated as “clean” and considered for forwarding. Low confidence predictions (confidence < 0.7) are routed for analyst verification by design. The ML pipeline also includes adversarial defenses, such as adversarial training, poisoning detection mechanisms, and input validation to reduce potential risk of model manipulation.

All the possible actions for each connection request are executed through a security orchestration, automation, and response (SOAR) engine server (case 7). A sample SOAR playbook is shown in Algorithm 1. For a “malicious” sample, the SOAR engine can automatically push the corresponding firewall rules to the gateway/proxy (EGW) (case 8) to block the attacker’s IP or IP ranges, revoke any associated certificates, update the edge blocklist, flag and quarantine the targeted device, create an incident ticket in the Security Operations Center (SOC), and notify engineers. For “suspicious” results, the playbook may throttle or quarantine the session at the middleware, restrict the implicated device’s operational privileges (for example, read-only mode or telemetry-only), and schedule human review. Clean requests are delivered to the IMWS (case 9) which performs final attestations (mutual TLS checks, device TPM attestation where available) through the IFW (case 10a) and forwards the message to the intended IoT target (case 10b). Importantly, the threat analysis results—new IoCs, updated detection rules, and model updates—are fed back to the gateway (EGW) (case 8) and honeypot farm through signed, secure channels for continuous learning from the TAS to the SEP (cases 11a and 11b).
**Algorithm 1** Sample SOAR playbook.name: honeypot-alert-playbooktrigger: honeypot.alertsteps:   - enrich_alert: threatintel.enrich(src_ip, domain, file_hash)   - score: ml.score(alert_features)   - decision:    if score >= 0.9:     - gw.block_ip(src_ip, duration=7d)     - revoke_cert(device_id)     - siem.create_incident(details)     - notify_soc(level=high)    elif 0.6 <= score < 0.9:     - middleware.quarantine_session(duration=1h)     - siem.create_incident(details)     - prompt_human_review()    else:     - forward_to_middleware()     - update_edge_rules(new_blocklist_entries)

Finally, in the case of an AIoT-based device attempts to access the internet, its outbound request does not go directly out but passes through a controlled security chain. Firstly, the IFW (case 12a) verifies if the connection request is permitted based on its protocol, destination, and predefined security policies. After this initial check, the request passes through the IMWS (case 12b) and EGW (case 12c), in which advanced inspection techniques, metadata analysis, and redirection security policies can be applied to identify potential suspicious activity. If the request is considered to be safe, the traffic is forwarded to the EFW (case 12d) that performs the final filtering and NAT operation, before allowing this to access the Internet (case 12e). During this process, MELT data are captured and may be forwarded to the CPS (case 3b2) for deeper behavior analysis.

## 5. Experimental Setup

### 5.1. Environmental Configurations

The experimental setup was deployed through a specialized virtual lab using a combination of local hosts, servers, and cloud infrastructure. Our testbed focuses on network-level and service-level attack surfaces, rather than low-level hardware or firmware vulnerabilities. Several empirical studies indicate that a significant portion of real-world IoT compromises occur at the service and protocol level, rather than through hardware-specific exploits. Accordingly, our evaluation focuses on modeling these prevalent attack surfaces.

Specifically, for the EGW, we deployed a dedicated VM running Envoy 1.35 as a standalone proxy [28] with proper custom edge scripts to define security policies. Also, we considered mutual TLS for both MQTT and HTTP traffic, as well as a basic rate limiting to control connection attempts. For suspicious flows, we implemented IP reputation scoring and shadowing, using thresholds such as more than 5 failed authentications within 60 s or more than 10 connection attempts per minute. Shadowed requests, including metadata and sanitized payloads, were forwarded to the Cloud Proxy for further inspection and analysis.

For the emulated IoT devices, we provisioned three Metasploitable 3 VMs (both Windows and Linux OS) [29] to represent vulnerable legacy devices and two lightweight Docker containers running Eclipse Mosquitto MQTT brokers 2.0.22 [9] with topic-level ACLs and mTLS, emulating common IoT middleware configurations. Metasploitable VMs are used as proxies for legacy or poorly maintained IoT gateways and edge devices, which are commonly observed in real deployments. To introduce protocol heterogeneity and emulate constrained IoT communication, a subset of devices exposed CoAP services over UDP, providing simple sensor and actuator endpoints. To emulate AIoT control logic, we integrated a Node-RED middleware layer that consumed MQTT telemetry streams and generated control commands and HTTP callbacks. This middleware acted as an application-level orchestration component between the emulated devices and the Edge Gateway, reflecting common AIoT data processing and control workflows. For the cloud deception layer, we deployed a low-interaction honeypot based on Cowrie 2.7.0 [30] to quickly capture brute-force login attempts and scanning behavior, and a high-interaction honeypot implemented as a containerized VM running a real device firmware image to capture in-depth exploit attempts, persistence mechanisms, and attacker post-exploitation activity. We placed all honeypots inside an isolated VPC/subnet with strict outbound rules so that connectivity from outside is permitted only to the SEP. For this (SEP), we implemented a Cuckoo-like dynamic analysis environment [31] that executed suspicious binaries and instrumented their runtime behavior, capturing artifacts such as system calls, network callbacks, and dropped files. We then converted those behavioral traces into feature vectors and forwarded them to the Threat Analysis Server (TAS) for correlation and scoring.

For the TAS, we used an ELK stack 9.1.5 (Elasticsearch, Logstash, and Kibana) [32] for centralized storage and visualization, together with a Python-based service, responsible for feature extraction and ML inference for decision making. The TAS ran an ensemble detection pipeline that combined Suricata signature matches [33], unsupervised anomaly detection (autoencoders trained on telemetry sequences), and supervised classification (XGBoost trained on labeled session-level features) [34]. Input features included failed login ratio, average packet size, inter-packet delay, payload entropy, unusual destination domains observed by the sandbox, number of spawned processes, YARA hits, and device context [35]. Therefore, the TAS produced a numeric maliciousness score in [0, 1] scale together with an explainability vector, indicating the top contributing features.

For the SOAR engine, we implemented a lightweight orchestration script that automated mitigation playbooks: pushing block rules to the EGW, quarantining or restricting implicated IoT devices through the middleware, and creating incidents in the SOC dashboard for analyst response. Finally, for the Cloud Proxy, we implemented a routing logic that accepted shadowed requests from the EGW and forwarded them either to the honeypot farm, for unknown or suspicious flows, or back to the middleware, for known and high-confidence clean traffic.

### 5.2. Real Data Collection

For baseline profiling, we generated 48 h of benign device traffic to capture normal AIoT behavior and bootstrap unsupervised models. The baseline emulated realistic device workloads and operational patterns across the entire testbed; the experiment used three Metasploitable VMs and two lightweight Docker device containers as telemetry sources. For this procedure we used these traffic patterns and workloads: periodic telemetry, MQTT publish/subscribe, occasional firmware checks and HTTP polling, and background management traffic.

Each emulated device published sensor readings at randomized intervals to mimic real devices. Inter-publish intervals were sampled from a uniform distribution between 5 and 30 s to create natural jitter and avoid perfectly periodic traces. Payloads contained small JSON objects (temperature, humidity, battery, timestamp) with realistic value ranges and occasional outlier values to reflect normal sensor noise.

The devices used MQTT over mTLS on port 8883. Each device subscribed to a small set of topics (e.g., device/<id>/telemetry, device/<id>/cmd) and published telemetry messages. We used a standard MQTT client (paho-mqtt) with randomized client IDs and quality of service (QoS) levels to reproduce typical IoT behavior. Also, the devices performed periodic HTTP(S) GET checks to a firmware update endpoint with low frequency (one check every 6–12 h per device), including standard headers and user-agent strings. Firmware check responses were emulated with benign reply sizes and timing. Additionally, occasional SSH/administration logins from trusted management hosts were simulated (successful logins only) to include benign authentication events in the baseline.

### 5.3. Implementation Process

The workload drivers were implemented as lightweight Python 3.13.7 scripts and docker containers that used paho-mqtt (a client class which enables applications to connect to an MQTT broker to publish messages, and to subscribe to topics and receive published messages) [36] for MQTT and requests for HTTP. Scripts were seeded with different random seeds per device to avoid synchronized behavior. Message payload sizes varied in a controlled range (typically 100–1200 bytes) to reflect telemetry versus update checks. All device clients used mTLS with test certificates issued by the lab Certificate Authority (CA), and certificate renewal events were simulated to be like a real-world environment.

The network packet captures (PCAPs) were recorded at three choke points using tcpdump 4.99.5 [37] and Zeek 8.0.1 network security monitor [38] for flow extraction and basic protocol parsing. PCAPs were ingested to an object store (S3-compatible) with server-side encryption enabled; sensitive fields that might contain private data like protected health information (PHI) were hashed before offsite upload. Data retention policy for raw PCAPs was enforced (configurable: default 90–180 days).

Time-series telemetry and aggregated flow metrics were sent into a central time-series database, InfluxDB [39], and indexed into Elasticsearch for search and visualization via Kibana 9.1.5. Logs were forwarded using Filebeat 9.1.5 and Logstash 9.1.5 with transport layer security (TLS) and mutual authentication [40].

For feature extraction and preprocessing, raw traces were transformed into session-level and time-windowed features using a reproducible pipeline: traffic was sessionized per (src_ip, dst_ip, protocol, dst_port) and aggregated into fixed windows (60 s and 5 min windows). For each session/window we computed network and behavioral features such as: average packet size, packet count, and byte totals; inter-packet time statistics (mean, median, variance); byte/entropy metrics of payloads (payload entropy, normalized entropy); session duration and number of distinct remote endpoints; authentication metrics (failed login ratio, successful login ratio, username variance); device context features (device model id, firmware version, last patch timestamp).

The sandbox/honeypot features were absent in baseline (no suspicious payloads executed) but the pipeline reserved fields so models and downstream systems had consistent schemas. The features were normalized (Min/Max or z-score) and low-variance features were dropped. We persisted preprocessed feature vectors in both the time-series DB for streaming experiments and as parquet/CSV artifacts for offline model training.

The baseline dataset was used to bootstrap unsupervised detectors such as autoencoders and one-class models. Autoencoders were trained only on benign windows to learn a nominal reconstruction distribution; reconstruction error thresholds were selected by analyzing the error distribution (set at mean + 3σ or via percentile). Cross-validation on hold-out benign windows was used to verify model stability and to choose hyperparameters (latent dims, learning rate, epoch count). We also injected a small, labeled set of synthetic anomalous windows (controlled faults, high failed-auth bursts) to validate that anomaly scores separated benign and anomalous behavior before any attacker traffic was introduced. All preprocessing, scaler parameters, and model checkpoints were versioned and archived (hash-signed) to support reproducibility and to detect training-time poisoning.

We should note that before any data left the local testbed (PCAPs or payloads), payload sanitization removed or hashed potential PHI and identifiers. Only sanitized payloads and metadata were forwarded to the analysis cloud. All stored artifacts were encrypted at rest and in transit. Access control (IAM roles and audit logging) was enforced for object store and Elasticsearch access. Retention policies were applied automatically to raw PCAPs and to derived artifacts consistent with GDPR considerations. Baseline collection ran entirely in the isolated lab network; no external IoT devices or third-party infrastructure were accessed during collection.

### 5.4. Experimental Procedure

The 48 h baseline produced a comprehensive profile of normal AIoT behavior, including per-device publish rates, authentication patterns, typical payload sizes, and timing distributions. These baseline features successfully bootstrapped the unsupervised models and provided stable normalization/scaling parameters used later in attack injections and streaming detection experiments.

Executed from a Kali attacker VM, three classes of attacks were simulated: (a) credential stuffing/brute-force against MQTT/SSH endpoints; (b) remote exploits targeting vulnerable device services with malicious payloads, and (c) staged recruitment: initial exploit followed by bot binary download and Command and Control (C2) beaconing attempts. Each scenario was repeated with varying attacker IPs, timings, and payloads to produce a diverse dataset. All attack traffic remained isolated in the lab environment; no external devices were impacted.

In addition to the traffic generated within our controlled virtual testbed, we leveraged the publicly available EdgeIIoTset dataset [41] for complementary offline training and validation of the supervised detection models. EdgeIIoTset focuses on network- and service-level IoT attacks, including brute-force, scanning, and denial-of-service behaviors, which align with the threat model considered in this work. The dataset was used to validate feature robustness and to assess generalization across heterogeneous traffic sources, while all real-time detection, mitigation, and SOAR performance metrics reported in this paper were derived exclusively from experiments conducted in our virtual lab.

## 6. Experimental Results

In our testbed, the EGW performed initial verification and filtering for every inbound connection: mutual TLS termination and certificate checks, rate limiting and basic IP-reputation score lookups. Flows that exceeded locally configured thresholds (for example, more than five failed authentication attempts within 60 s or more than ten connection attempts per minute from the same source) were shadowed—that is, a sanitized copy containing metadata and an anonymized payload was created—and forwarded to a Cloud Proxy for further inspection. The Cloud Proxy applied routing logic and directed suspicious flows into the honeypot farm. Low-interaction honeypots were used to rapidly capture brute-force and scanning behavior, while high-interaction honeypots (containerized VMs running real device firmware) captured deeper exploit attempts and persistence mechanisms. Files and suspicious payloads harvested by the honeypots were forwarded to an isolated sandbox for dynamic analysis; the sandbox executed samples in a controlled environment and returned behavioral vectors (syscalls, network callbacks, and dropped files) to the TAS. The TAS performed automated feature extraction from telemetry, honeypot and sandbox artifacts and applied an ensemble ML pipeline to compute a numeric maliciousness score for each flow.

Decisions were made according to fixed score thresholds. These thresholds were empirically selected for this experimental setup and were not optimized for adversarial adaptation or long-term changes in underlying data patterns. Scores ≥ 0.9 were labeled “malicious” and triggered immediate, automated mitigation via the SOAR engine (for example: push a block rule to the gateway, revoke active sessions, and quarantine the implicated device). Scores in the [0.6, 0.9) range were labeled “suspicious”; these flows were quarantined at the middleware and escalated for human analyst review. Scores < 0.6 were treated as clean and forwarded to the internal middleware server for final attestations and delivery to the target IoT device. All actions were logged and correlated for auditability.

We collected a comprehensive dataset consisting of PCAPs from honeypots, sandbox logs, ELK-ingested telemetry from the TAS and gateway, SOAR action logs, and gateway metrics (e.g., requests per second, rejected requests). Performance was evaluated using operational metrics including mean-time-to-detect (MTTD), mean-time-to-mitigate (MTTM), true positive rate (TPR), false positive rate (FPR), added latency for clean flows (measured as client round trip time), and cloud bandwidth consumption for escalated traffic.

In the controlled lab experiments the combined system achieved median MTTD on the order of tens of seconds for active brute-force and exploit attempts (with faster detection for noisy brute-force activity captured by low-interaction honeypots and slower detection for stealthy, low-and-slow anomalies detected by the autoencoder). Median MTTM for fully automated malicious detections was under two minutes (including pushing a block rule and quarantining the device); suspicious cases requiring human review incurred longer mitigation times, as expected. Importantly, the edge-filtering strategy kept latency overhead for clean, high-confidence flows minimal (median increase < 30 ms), and only about 8–12% of total connections during attack windows were escalated to the cloud for full analysis, validating the approach within the scope of the evaluated experimental setup, conducted under a controlled testbed with predefined workloads and traffic patterns.

**Figure 2:**
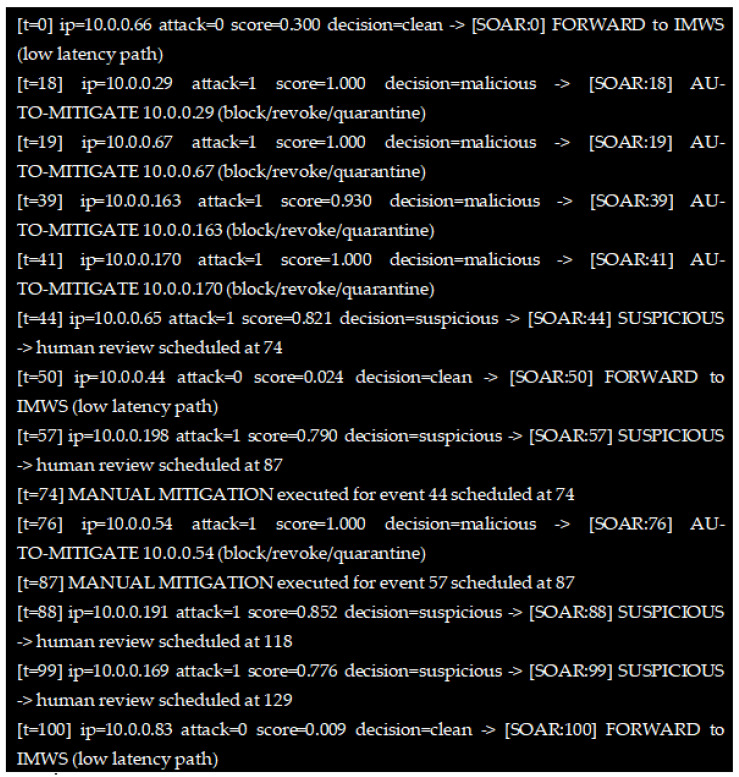
Sample connection events processed by the EGW and TAS during the experiment.

Figure 2 illustrates a sequence of sample connection events processed by the EGW and TAS. Each event is annotated with a timestamp [t], source IP, attack label, computed maliciousness score, and the corresponding decision. For clean traffic (low score), the SOAR engine forwards the request to the IMWS along the low-latency path. For malicious traffic (high score), the SOAR engine enables automated mitigation actions, including IP blocking, certificate revocation, and device quarantine.

Table 3 shows sample outcomes from the honeypot-alert SOAR playbook. Each row corresponds to an inbound session, with ML-derived scores and the final decision, according to the Algorithm 1. Malicious sessions (score ≥ 0.9) triggered automated mitigation, suspicious sessions (0.6 ≤ score < 0.9) were quarantined and flagged for human review, and clean sessions were forwarded to the middleware for normal delivery.

**Figure 3:**
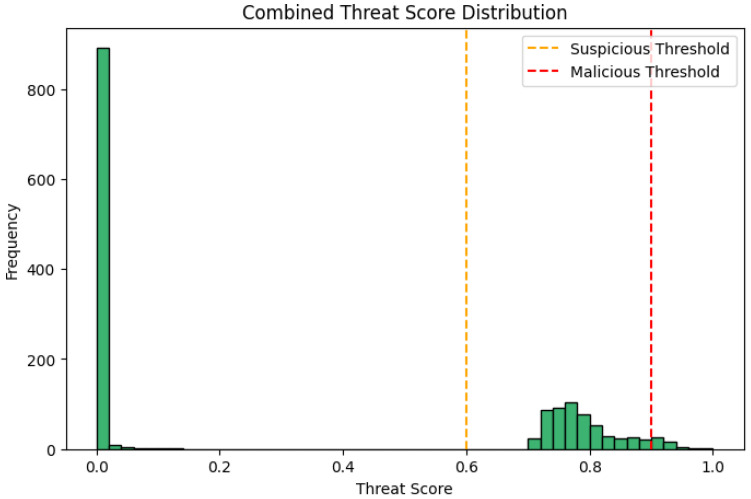
Combined threat score distribution of the sample events from an experimental run representative of the overall results. The green bars show the distribution of combined threat scores across all events.

**Figure 4:**
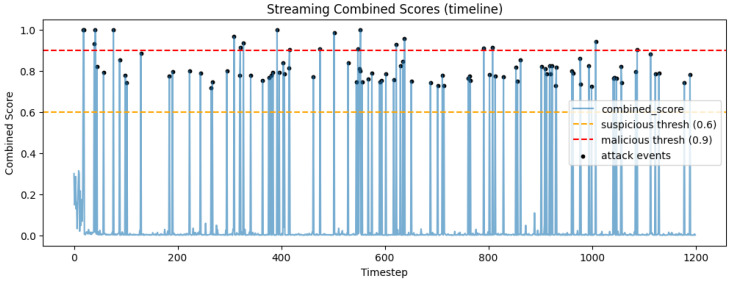
Streaming combined scores of the sample events from an experimental run representative of the overall results.

**Figure 5:**
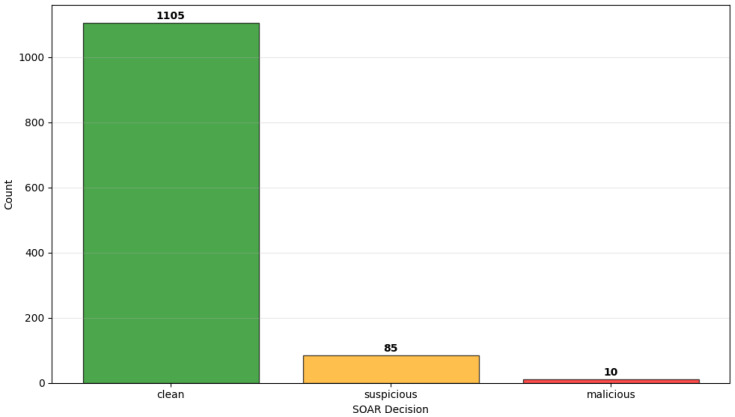
Decision counts according to the SOAR playbook (Algorithm 1) from an experimental run representative of the overall results.

Finally, the simulated traffic stream completed in a total time of 120.28 s, processing 1200 events. Out of these, as it is shown in Figure 3 and Figure 4, 96 were attack events (8% of the total sample). As shown in Figure 5, 95 of the 96 events were successfully detected, and only 1 was not detected as malicious or suspicious by the SOAR. In total, 10 attacks of 95 (10.53%) received automated mitigations, while the rest of these attacks labeled as suspicious and enabled SOAR for decision. Overall, the stream-level evaluation demonstrates very high detection performance for the evaluated attack scenarios, with a true positive rate (TPR) (Recall) of 0.9896, a false negative rate (FNR) of 0.0104, a true negative rate (TNR) of 1.000, and a false positive rate (FPR) of 0.0000 during the conducted experiments, which primarily involved structured benign workloads and scripted attack behaviors. The positive predictive value (PPV) (Precision) is 1.000, while the accuracy is 0.9992, and the F1-score is 0.9948. These results confirm the effectiveness of the proposed architecture under the specific experimental assumptions and threat models considered in this research study.

In this point, it should be noted that the presented evaluation was conducted in a controlled virtual testbed, which although it is representative under specific circumstances (streaming evaluations spanning 120 s windows and a 48 h baseline for profiling normal behavior), it does not fully simulate the complexity, scale, and unpredictability of real-world AIoT deployments. The attack scenarios were executed using specific tools and scripts, rather than adaptive human-driven adversaries capable of evolving strategies in response to defensive actions. As a result, the reported detection metrics should be interpreted as an optimistic—but informative—upper bound on performance under controlled conditions, rather than as a direct proxy for real-world deployment performance. In other words, the evaluation does not assess robustness under adversarial adaptation, long-term evolution of device behavior and network traffic, or performance variability across heterogeneous AΙοΤ deployments, which remain important directions for future work.

## 7. Comparative Analysis

In this section, we present a comparative analysis with the most related and state-of-the-art schemes to highlight the performance and effectiveness of our proposed model. Specifically, we chose four research works from Table 1 of the Related Work section, as follows: Elijah et al. [5], Menon et al. [6], Bibi et al. [10], and Latesh Kumar et al. [12].

While these prior works focus primarily on detection accuracy or architectural considerations in isolation, they lack an integrated, closed-loop architecture that combines edge-level detection, deception-based intelligence, sandbox-driven analysis, explainable scoring, and automated mitigation. Our proposed architecture addresses this gap by integrating edge-level detection, protocol-aware middleware, deception-based intelligence, and automated response into a single, operational AIoT security pipeline. Specifically, it performs early traffic inspection and policy enforcement at the EGW, selectively shadows suspicious flows toward a cloud-based deception layer, and executes captured payloads in a sandboxed environment to extract behavioral indicators. These indicators are fused with network- and application-level features by the TAS, which produces both a quantitative maliciousness score and an explainability vector. Finally, a lightweight SOAR component closes the loop by automatically triggering mitigation actions—such as blocking, device quarantine, and incident creation—while preserving privacy through payload sanitization and strict data governance controls.

**Figure 6:**
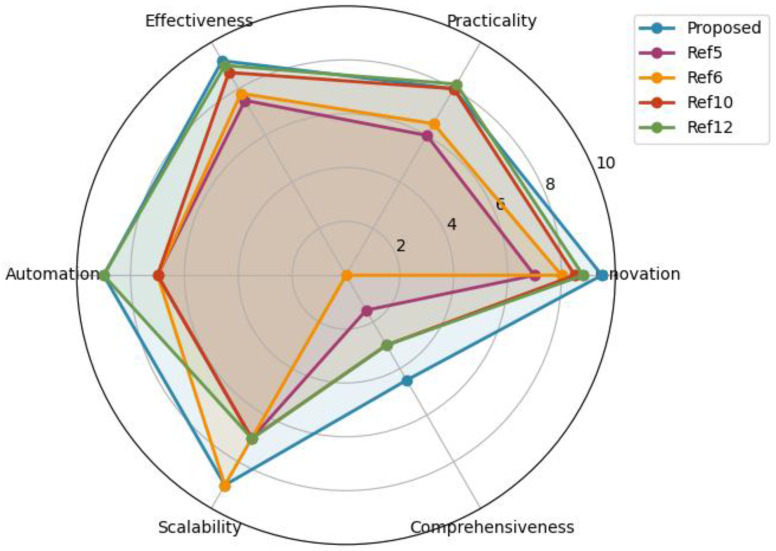
Qualitative radar-chart comparison of the proposed AIoT security architecture against representative state-of-the-art approaches.

Figure 6 indicates a qualitative comparative assessment of our proposed system compared to the above state-of-the-art schemes in terms of practicality, innovation, comprehensiveness, scalability, automation, and effectiveness. Scores were assigned on a 0–10 ordinal scale based on the presence, maturity, and integration level of each capability as explicitly reported in the corresponding publications, following a consistent rubric across all compared systems. It should be noted that the assessment does not reflect absolute performance metrics, but relative architectural coverage. As illustrated in this figure, our proposed architecture exhibits broader coverage across key AIoT security dimensions, particularly in automation, comprehensiveness, and scalability. Unlike prior works that emphasize detection accuracy in isolation, our approach integrates early detection, deception-based intelligence, forensic analysis, and automated mitigation within a single operational pipeline.

It should be emphasized that the comparison presented in Figure 6 does not aim to benchmark detection accuracy or absolute performance metrics. Instead, it evaluates the proposed architecture completeness and its operational capabilities, which are not jointly addressed in other related research works.

## 8. Conclusions and Future Work

A novel architecture for mitigating cyber threats in AIoT environments was presented. The main idea of our proposed work was to design and implement a novel hybrid architecture for hardening AIoT environments against botnet attacks by combining edge-based filtering, cloud-hosted honeypots, sandboxing, and ML–driven threat analysis of the connection requests. Incoming requests from external clients were first processed at a sophisticated IoT gateway, which filtered traffic and redirected suspicious flows to a cloud deception layer where honeypots mirrored real devices of the AIoT environment to capture potential malicious activities. Payloads and artifacts were analyzed in an intelligent sandbox, empowered with ML to classify the traffic using explainable features to support SOC decisions. Automated mitigation actions were executed through a lightweight SOAR engine server, while benign traffic was safely routed to the AI-based IoT devices. Experimental validation in an isolated and controlled lab highlighted that this architecture achieved rapid detection and protection with a low mean time to detect and low mean time to mitigate, while maintaining minimal latency for normal operations. These results indicate the feasibility and effectiveness of the proposed approach as a proof-of-concept, though they do not fully capture the complexities of real-world deployments.

Future work could improve the scalability and resilience of our proposed model by integrating FL at the EGW in the DMZ, thereby avoiding the sharing of the raw MELT data. This perspective could expand the honeypot diversity by utilizing suitable container orchestration platforms like Kubernetes and applying hardware-based verification mechanisms to prevent compromises, such as unauthorized access, data leakage, or system tampering. Additional improvements could include advanced adversarial ML defenses, continuous red/blue team testing with real honeypot captures, and extended SOAR playbooks with automated recovery workflows. These efforts will help to further evaluate the system in larger, heterogeneous, and real-world AIoT environments. A comparative analysis with other related state-of-the-art architectures constitutes an additional direction for future research. While physical compromise and hardware-level attacks are important aspects of IoT security, this work intentionally focuses on network- and application-layer threats. Extending the evaluation to hardware-level attacks [42] is part of our planned future work.

## Figures and Tables

**Table 1:** Comparison of proposed architecture with related AIoT IDS frameworks.

Study/Framework	Architecture Level	Detection Technique	Targeted Threats	Protocols/Devices	Edge/Cloud	Explainability/Analytics	Notes/Strengths
Proposed Model	Edge–Cloud (3-layer: devices, middleware, EGW, cloud)	Ensemble ML (Suricata, Autoencoders, XGBoost), anomaly detection	Credential stuffing, remote exploits, bot recruitment	MQTT (Mosquitto, ACLs, mTLS), CoAP, HTTP, SSH	Edge inference, cloud analysis	Feature-level explainability, dynamic scoring	Emulates heterogeneous AIoT deployments, realistic middleware, constrained device behavior
Elijah et al. [5]	AI-driven NGN/Edge–Cloud	ML, DL, hybrid ensembles	Botnet, malware, DDoS	IoT, edge traffic	Federated frameworks	Emphasis on privacy, adversarial robustness	Highlights dataset gaps, emphasizes lightweight models
Menon et al. [6]	Tri-level AIoT stack (edge, fog, cloud)	ML/DL (supervised/unsupervised/RL)	Authentication, DDoS, malware	Heterogeneous IoT	Edge, cloud, fog	Interoperability, explainability, continual learning	Focus on multi-layer architecture, RL for dynamic policies
Hassan et al. [7]	Layered IoT (perception, network, transport, application, business)	Hybrid ML/DL, RL	Jamming, spoofing, Sybil/MITM, malware	IoT stacks	Edge, cloud	Context-aware, end-to-end controls	Cross-layer risk analysis, supply-chain aware
Afrin et al. [8]	Distributed AIoT (GRC focus)	ML/DL, privacy-preserving analytics (FL, DP)	IoT security incidents	IoT pipelines	Edge, cloud	Auditability, model accountability	Focus on lifecycle governance, compliance
Wang et al. [9]	IoT traffic-focused IDS	DL (CNN, LSTM, hybrid), traditional classifiers	Label scarcity, anomaly detection	IoT devices	Edge inference vs. cloud offloading	Benchmarking, feature analysis	Streaming minibatch updates, microcontroller-friendly
Bibi et al. [10]	Edge-centric IDS	CNN/LSTM hybrids + flow stats	Volumetric, protocol DDoS	IoT traffic	Edge gateways, cooperative signals	Adaptive thresholds (RL)	Quick triage, local mitigation, robustness to traffic mutation
Mishra [11]	Integrated IoT stack	DL-based anomaly detection, policy enforcement	Anomaly detection, key revocation	Restricted IoT devices	Edge/offload	Explainability, federated retraining	Modular design, balances latency, energy
Latesh Kumar et al. [12]	Cloud-based threat prediction	CorrNet (correlational RNN autoencoder)	Multi-source anomalies, VM attacks	Cloud services	Cloud	Forensic capabilities, cross-reconstruction error analysis	Combined AI, traditional tools, validated on campus gateway

**Table 2:** Main architectural components and roles.

Component	Description
Edge Gateway (EGW)	Intelligent inspection layer in the DMZ that performs mTLS/CRL validation, protocol sanity checks, rate limiting, telemetry collection, and dynamic security enforcement for incoming and outgoing traffic.
Honeypot Farm	A two-tier deception layer (low- and high-interaction honeypots) deployed in an isolated VPC, emulating real IoT devices to capture attacker behavior, exploits, and payloads.
Sandbox Endpoint (SEP)	Executes suspicious binaries and commands captured from honeypots in a controlled environment to analyze dynamic behavior, system changes, and network callbacks.
Threat Analysis Server (TAS)	Correlates edge telemetry, honeypot data, and sandbox outputs using hybrid detection models (signature-, rule-, and ML-based) to generate threat scores and behavioral insights.
SOAR Engine	Automates incident response actions (e.g., blocking, quarantining, certificate revocation), orchestrates mitigation workflows, and feeds decisions back to the gateway and analysis pipeline.
SIEM (NGSIEMS)	Collects, aggregates, and visualizes logs, alerts, and incidents from all components, supporting centralized monitoring, forensic analysis, and threat intelligence integration.

**Table 3:** A sample of 10 outcomes from the honeypot-alert SOAR playbook.

Timestep	Client_ip	Is_Attack_gt	Sup_Score	Recon_Err	Combined_Score	Decision
18	10.0.0.29	1	1.000000	0.055525	1.000000	malicious
19	10.0.0.67	1	1.000000	0.087973	1.000000	malicious
39	10.0.0.163	1	1.000000	0.067324	0.929585	malicious
41	10.0.0.170	1	1.000000	0.202252	1.000000	malicious
44	10.0.0.65	1	0.953333	0.103813	0.821319	suspicious
57	10.0.0.198	1	1.000000	0.060960	0.790421	suspicious
76	10.0.0.54	1	1.000000	0.313014	1.000000	malicious
88	10.0.0.191	1	1.000000	0.158750	0.852150	suspicious
99	10.0.0.169	1	1.000000	0.079615	0.776305	suspicious
102	10.0.0.33	1	1.000000	0.043754	0.741935	suspicious

## Data Availability

Data are contained within the article.

## Abbreviations

The following abbreviations are used in this manuscript:

5G	5th Generation
6G	6th Generation
AE-CNN	AutoEncoder Convolutional Neural Network
AI	Artificial Intelligence
AIoT	Artificial Intelligence of Things
API	Application Programming Interface
BoT	Botnet of Things
C2	Command and Control
CA	Certificate Authority
CCPA	California Consumer Protection Act
CNN	Convolutional Neural Network
COAP	Constrained Application Protocol
CorrNet	Correlational Recurrent Neural Network
CPS	Cyber Physical Systems
CRL	Certificate Revocation List
CSV	Comma-Separated Values
DB	Database
DBN	Deep Belief Network
DDoS	Distributed Denial of Service
DL	Deep Learning
DMZ	Demilitarized Zone
DNS	Domain Name System
DPI	Deep Packet Inspection
DRL	Deep Reinforcement Learning
DT	Decision Tree
EFW	External Firewall
EGW	Edge Gateway
ELK	Elasticsearch, Kibana & Logstash
EtherCAT	Ethernet for Control Automation Technology
FL	Federated Learning
FNR	False Negative Rate
FPR	False Positive Rate
GAN	Generative Adversarial Network
GDPR	General Data Protection Regulation
GeoIP	Geographical IP
GPS	Global Positioning System
GRC	Governance, Risk, and Compliance
HTTP	HyperText Transfer Protocol
HTTPS	Hypertext Transfer Protocol Secure
IAM	Identity and Access Management
ID	Identity Document
IDPS	Intrusion Detection and Prevention System
IDS	Intrusion Detection System
IFW	Internal Firewall
IMWS	Internal Middleware Server
InfluxDB	Influx Database
IoC	Indicators of Compromise
IoT	Internet of Things
IP	Internet Protocol
JSON	JavaScript Object Notation
KNN	K-Nearest Neighbors
LSTM	Long Short-Term Memory
MELT	Metrics, Events, Logs, and Traces
MitM	Man-in-the-Middle
ML	Machine Learning
Modbus	Modicon Bus
MQTT	Message Queuing Telemetry Transport
mTLS	mutual Transport Layer Security
MTTD	Mean Time To Detect
MTTM	Mean Time To Mitigate
NAT	Network Address Translation
NGN	Next-Generation Network
NGSIEMS	Next Generation Security Information and Event Management
Nmap	Network Mapper
OPC-UA	Open Platform Communications—Unified Architecture
OS	Operating System
PCAP	Packet Capture
PHI	Protected Health Information
PLC	Programmable Logic Controller
PPV	Positive Predictive Value
PROFINET	Process Field Network
QoS	Quality of Service
RBM	Restricted Boltzmann Machine
RF	Random Forest
RL	Reinforcement Learning
RNN	Recurrent Neural Network
SDN	Software-Defined Networking
SEP	Sandbox Endpoint
SIEM	Security Information and Event Management
SOAR	Security Orchestration, Automation, and Response
SOARES	Security Orchestration, Automation, and Response Engine Server
SOC	Security Operations Center
SSH	Secure Shell
SVM	Support Vector Machine
TAS	Threat Analysis Server
TLS	Transport Layer Security
TNR	True Negative Rate
TPM	Trusted Platform Module
TPP	Threat Prediction and Protection
TPR	True Positive Rate
VM	Virtual Machine
VPC	Virtual Private Cloud
XAI	Explainable Artificial Intelligence
XGBoost	eXtreme Gradient Boosting
YARA	Yet Another Ridiculous Acronym
